# Interleukin-6-elicited chronic neuroinflammation may decrease survival but is not sufficient to drive disease progression in a mouse model of Leigh syndrome

**DOI:** 10.1186/s12950-023-00369-4

**Published:** 2024-01-11

**Authors:** Kevin Aguilar, Carla Canal, Gemma Comes, Sandra Díaz-Clavero, Maria Angeles Llanos, Albert Quintana, Elisenda Sanz, Juan Hidalgo

**Affiliations:** 1 Department of Cellular Biology, Physiology and Immunology, Animal Physiology Unit, Faculty of Biosciences, Universitat Autònoma de Barcelona, Bellaterra, Barcelona, Spain 08193; 2 Institut de Neurociències, Universitat Autònoma de Barcelona, Bellaterra, Barcelona, Spain; 3 Present address: Institute for Research in Biomedicine (IRB Barcelona), Barcelona, Spain; 4 Present address: Dementia Research Institute, Imperial College London, London, UK

**Keywords:** Leigh syndrome, *Ndufs4 KO*, GFAP-IL6, Neuroinflammation, Microglia, Astrocytes, IL-6

## Abstract

**Background:**

Mitochondrial diseases (MDs) are genetic disorders characterized by dysfunctions in mitochondria. Clinical data suggest that additional factors, beyond genetics, contribute to the onset and progression of this group of diseases, but these influencing factors remain largely unknown. Mounting evidence indicates that immune dysregulation or distress could play a role. Clinical observations have described the co-incidence of infection and the onset of the disease as well as the worsening of symptoms following infection. These findings highlight the complex interactions between MDs and immunity and underscore the need to better understand their underlying relationships.

**Results:**

We used *Ndufs4 KO* mice, a well-established mouse model of Leigh syndrome (one of the most relevant MDs), to test whether chronic induction of a neuroinflammatory state in the central nervous system before the development of neurological symptoms would affect both the onset and progression of the disease in *Ndufs4 KO* mice. To this aim, we took advantage of the GFAP-IL6 mouse, which overexpresses interleukin-6 (IL-6) in astrocytes and produces chronic glial reactivity, by generating a mouse line with IL-6 overexpression and NDUFS4 deficiency. IL-6 overexpression aggravated the mortality of female *Ndufs4 KO* mice but did not alter the main motor and respiratory phenotypes measured in any sex. Interestingly, an abnormal region-dependent microglial response to IL-6 overexpression was observed in *Ndufs4 KO* mice compared to controls.

**Conclusion:**

Overall, our data indicate that chronic neuroinflammation may worsen the disease in *Ndufs4 KO* female mice, but not in males, and uncovers an abnormal microglial response due to OXPHOS dysfunction, which may have implications for our understanding of the effect of OXPHOS dysfunction in microglia.

**Supplementary Information:**

The online version contains supplementary material available at 10.1186/s12950-023-00369-4.

## Introduction

 Mitochondrial diseases (MDs) are rare genetic disorders caused by mutations in genes essential for proper mitochondrial function. Since the first mutations were described [1, 2], more than 350 causal mutations in nuclear DNA (nDNA) or mitochondrial DNA (mtDNA) have been identified. This enormous plethora of mutations gives rise to a wide range of heterogeneous diseases that vary in disease onset, prognosis, and tissue susceptibility [3]. The large number of disease etiologies that involve different defects in mitochondrial biology ultimately affecting oxidative phosphorylation (OXPHOS), partially explains the high degree of heterogeneity and difficulty in establishing effective treatments [4]. In this type of disease, it is the central nervous system (CNS) and muscles the most affected tissues since they have elevated energy requirements that rely on correct OXPHOS function, nonetheless, MDs can encompass dysfunction of any organ or tissue [3, 5].

During the past decade, substantial knowledge has been obtained regarding the pathological mechanism that underlie mitochondrial diseases [4, 6, 7]. In recent years, there has been a growing interest in immune-related processes. As organelles with a bacterial origin, mitochondria possess a repertoire of molecules that can be detected by pattern recognition receptors (PRRs). In particular, mtDNA and mtRNAs have a strong resemblance to those of bacterial origin and can trigger both antiviral and bacterial-like innate immune responses [8, 9]. In a context of mitochondrial stress, damage, or dysfunction, mtDNA and mtRNAs can escape the mitochondrial matrix, be released to the cytoplasm, and engage an inflammatory response via the inflammasome, TLR9, mitochondrial antiviral signaling proteins (MAVS), and/or cGAS-STING pathways [10–12]. Noteworthy, these signaling networks allow explaining why throughout many severe disabling diseases, mitochondrial malfunction, degeneration, and persistent inflammation occur together. Therefore, it has been speculated that the mitochondria-innate immune crosstalk contributes to the pathobiology of MDs [11]. One example of this crosstalk is observed in polymerase gamma (POLG) mutator mice, a mouse model of mtDNA instability that mirrors some clinical features of patients with POLG-related MD and aging [13]. In these mice, aged individuals display chronic cGAS-STING-dependent interferon type-I (IFN-I) signaling in multiple organs, including the heart, liver, kidney, and an expansion of the myeloid cell population [14]. Additionally, in mouse models of mitochondrial-related myopathies, mtDNA has been shown to promote inflammation and muscle atrophy [15, 16]. Furthermore, recent studies have highlighted the emerging role of microglia and peripheral immune cells in the development of the *Ndufs4 KO* encephalopathy, a mouse model of Leigh syndrome (LS) [17–19]. Immune-related processes have not only been speculated to directly guide the pathology in MDs, but also to influence their onset and their degree of progression [20, 21]. Some forms of MDs can manifest prenatally or during the early stages of life, but a considerable number of patients may remain asymptomatic until adulthood. Furthermore, even those from childhood-onset usually do not exhibit symptoms at birth [3, 7]. Importantly, not only can different mutations result in different clinical features, but also the same mutation might lead to different disease onset and severity [22–25]. All these pieces of evidence suggest that additional factors, apart from the specific genetic mutation, could contribute to the onset and development of the pathology. Currently, the factors that can trigger the onset and influence the progression of mitochondrial diseases are unknown. Despite this lack of knowledge, clinical evidence in patients suffering from different types of MDs indicates that immune dysregulation or immune distress could be important players based on: (i) the concurrence of symptoms with infection, where the presence of an infection may trigger the onset of the disease and (ii) the overlap of infection, periods of deterioration, and mental regression, which, at the same time, coincides with the worsening of the symptoms either during or after the infection [21]. Altogether, these observations highlight the complex interactions between mitochondrial diseases and the immune system.

Mice lacking the subunit NDUFS4 of the complex I (CI) of the electron respiratory chain, known as *Ndufs4 KO* mice, develop a fatal fast-progressing encephalopathy and die approximately at the postnatal day 55. Importantly, the disease present in *Ndufs4 KO* closely resembles the one found in patients with Leigh syndrome. Leigh syndrome (OMIM 25,600), also referred to as subacute necrotizing encephalopathy, is a mitochondrial disease that has an estimated prevalence of 1 case per 40,000 individuals [26]. Usually, disease onset starts at 2 years of age after an initial period of normal development followed by a physiological challenge (i.e., infection, surgery) and has a fatal prognosis with a mean survival of 2 years after the first symptoms [27]. Given the growing evidence indicating that immune/inflammatory dysregulation could trigger and exacerbate mitochondrial diseases, we aimed to assess how the chronic induction of a neuroinflammatory state during the presymptomatic phase of *Ndufs4 KO* mice would impact the onset and progression of the disease in these animals. For that purpose, we utilized GFAP-IL6 mice, which are a commonly used model for chronic neuroinflammation, and NDUFS4-deficient mice to generate *Ndufs4 KO* mice with chronic neuroinflammation (GFAP-IL6/*Ndufs4 KO*). Specifically, GFAP-IL6 mice overexpress the cytokine interleukin-6 (IL-6) under the control of the *Gfap* promoter, limiting the overexpression to astrocytes [28]. As IL-6 can act as a strong pro-inflammatory cytokine in the brain [29], GFAP-IL6 mice exhibit early microgliosis and astrogliosis, with both cell types displaying reactive morphological features [28, 30]. We found that the phenotype of female *Ndufs4 KO* mice was somewhat exacerbated by IL-6 overexpression at the survival level, while that of male mice remained unaffected. In addition, the motor decline was similar between *Ndufs4 KO* mice and GFAP-IL6/*Ndufs4 KO* regardless of sex. Notably, *Ndufs4 KO* mice showed an atypical microglial response to IL-6 overexpression compared to the control group. Altogether, our data suggest that the chronic increase in brain inflammation through overexpression of IL-6 had a limited effect on the progression of the disease in *Ndufs4 KO* mice and that CI dysfunction may compromise to some extent microglial response to IL-6 stimulation, and potentially to other inflammatory stimuli.

## Materials and methods

### Mice

All mice used in this study were kept in a 12-hour light-dark cycle under specific pathogen-free conditions at constant temperature (22 ± 2 °C) and fed standard chow diet and water *ad libitum*.

### Mice generation

The parental mouse strains used in this study were *Ndufs4*^*+/−*^ (MGI:5,614,092) [31], and GFAP-IL6 mice (MGI:7,327,600) [28]. All mouse lines were backcrossed with a C57BL/6 background for at least ten generations. Both male and female mice were used in the present study. Two rounds of breeding were required to generate NDUFS4 deficient mice with the astrocytic-targeted IL-6 overexpression. Firstly, GFAP-IL6 mice were crossed with *Ndufs4*^*+/−*^ mice. From the resulting offspring, *Ndufs4*^*+/−*^ and *GFAP-IL6/Ndufs4*^*+/−*^ mice were crossed, producing six different genotypes; four of them were considered for this study: control (*Ndufs4*^*+/−*^), GFAP-IL6 (*GFAP-IL6/Ndufs4*^*+/−*^), *Ndufs4 KO* (*Ndufs4*^*−/−*^), and GFAP-IL6/*Ndufs4 KO* (*GFAP-IL6/Ndufs4*^*−/−*^). IL-6 overexpression was conserved in hemizygosis since mice harbouring two copies of the transgene have a severe motor disorder at early ages [28].

### Genotyping

Mice were genotyped by PCR using DNA extracted from their tails. For DNA extraction, the tails were boiled in 100 µL of 50 mM NaOH solution for 7 min. The following primers were used: 5’-AGCCTGTTCTCATACCTCGG-3’, 5’-GTCCTCTATGAGGGTACAGAG-3’, and 5’-GGTGCATACTTATACTACTAGTAG-3’ for *Ndufs4* genotyping; 5’-GATCCAGACATGATAAGATA-3’ and 5’-CCGAAAAAACTCGGAATGG-3’ for IL-6 overexpression.

### Clinical evaluation

During the study, mice were weighed and examined daily or every other day for clinical signs (ataxia, and gait/postural alterations). Paw clasping behavior and body twisting were examined by suspending the animals by the tail for 20 s and measured as the presence or absence of the behavior; the day of onset was recorded. Food pellets and hydrogel were placed on the cage floor when *Ndufs4 KO* mice start to present paw paralysis. Finally, mice were humanely euthanized after a 20% loss of maximum body weight, after veterinary recommendation, or when they were found moribund. Importantly, these euthanasia criteria contributed to variations in group sizes across the different measures. Disease staging of NDUFS4-deficient mice was performed following available published guidelines [32] as previously described [17]. Briefly, disease progression was divided in early stage (P0–P29), mid stage (P30–41), and late stage (over P41).

### Behavioral tests

Mice were tested at postnatal days P27–29, P38–39, and P48–49 corresponding with early, mid, and late stages, respectively. All mice were tested after habituation to the testing room (30 min). All behavioral tests were conducted during the light phase (8 am – 1 pm). Due to the extremely narrow temporal window, some of the animals included in the experiments could not perform all tests at all ages. Nevertheless, efforts were made to avoid this situation as much as possible. Genotype identities were not blinded during behavioral testing.

### Rotarod

Accelerating rotarod was used to monitor motor coordination and balance. Mice were placed in a rotating bar that accelerated from 4 rpm to 40 rpm in a 5-min lapse (Harvard Apparatus, Holliston, MA, USA). The test was performed thrice with a minimum lapse of 10 min between trials. Latency to fall from the rod was recorded and reported as the average across the three trials. All mice underwent one training session before the first rotarod test.

### Open field (OF)

Mice were placed in a rectangular white methacrylate white box (36.5 × 56 × 31 cm) and allowed to freely explore the apparatus for 10 min. The total ambulation distance was measured. The activity of the mice was recorded through videography by an overhead video camera. All tests recorded were analyzed using EthoVision XT tracking software (Noldus Information Technology bv., Wageningen, the Netherlands).

### Whole-body plethysmography

Respiratory function was assessed using an unrestrained whole-body plethysmography. Detailed protocol can be found elsewhere [33]. In short, mice were given a 45-min period to adjust to the plethysmograph chamber (EMMS, ref. PLY310), after which a 15-min experimental period was conducted. The study reports the normalized tidal volume (which refers to the volume of air that is moved in or out of the lungs during a normal breath) per body weight (in µL·g^−1^), as well as the respiratory frequency (measured in breaths·min^−1^).

### Multiplex

For cytokine level detection in brain tissue, euthanasia of mice was conducted through decapitation at P38 for mid-stage and between P47-P55 for late-stage specimens. The brain regions were subsequently extracted, flash-frozen in liquid nitrogen, and stored at -80 °C until further usage. The multiplex analysis for brain tissue was previously optimized and validated in our laboratory [34]. To briefly summarize, both olfactory bulbs (OBs), half cerebellums, and half cortex hemispheres were mechanically homogenized using an MM-400 mixer mill (Retsch GmbH, Haan, Germany), followed by ultrasonic homogenization using Sartorius-LABSONIC P (Sartorius AG, Göttingen, Germany). The samples were homogenized in 100 µl (OB), 250 µl (cerebellum), and 300 µl (cortex) of ice-cold protein extraction buffer. Every 10 milliliters of homogenization buffer comprised 2.5 mL of 25 mM HEPES, 20 µl of 10% IGEPAL, 0.5 ml of 0.1 M MgCl2, 130 µl of 1.3 mM EDTA (pH 8.0), and 100 µl of 0.1 M EGTA (pH 8.0). The homogenization buffer was supplemented with a 1% protease inhibitor cocktail and 0.1 M phenylmethylsulphonyl fluoride (Sigma-Aldrich P8340 and P7626, respectively). Following sonication, the homogenate was centrifuged at 12,000 g for 5 min, and the supernatant was collected to determine the protein concentration using the PierceTM BCA Protein Assay (Thermofisher ref. 23.227). A protein concentration of 7–12 µg/µl of the tissue lysate was used to quantify the abundance of IL-6, TNF-α, IL-10, and IL-1β using the Mouse High Sensitivity T Cell Magnetic Bead Panel (Millipore, ref. MHSTCMAG-70 K) following the manufacturer’s instructions. Data were obtained using a Luminex MAGPIX instrument system (Luminex Corporation, Austin, TX, USA) and analyzed using the xPONENT software v4.2 (Luminex Corporation, Austin, TX, USA). All results were normalized to the protein concentration in the lysate. The levels of IL-1β were not quantifiable as the values seemed to be below the detection threshold of the technique.

### Tissue preparation for immunostaining

Mice were euthanized by decapitation at P38–39 and P48–P55 for mid and late stages, respectively. Brains were fixed in 4% paraformaldehyde (PFA) solution at 4 °C for 24 h and then transferred to cryoprotective 30% sucrose in PBS (Phosphate-buffered saline) (0.01 M pH 7.4) solution for 48 h at 4 °C. The entire brain was preserved for immunostaining. Before freezing, olfactory bulbs (OB) were separated from the rest of the brain by cutting coronally the prefrontal cortex. OBs were embedded in OCT medium and frozen in dry ice, whereas the remaining brain was frozen by immersion in isobutane at -30 °C. All brains used for immunostaining were kept at -80 °C until sectioning.

20 μm coronal sections of the brain were obtained using a Leica CM3050 S cryostat (Leica Biosystems GmbH, Wetzlar, Germany). Brains were sectioned from bregma 0.62 mm to -2.46 mm, and from bregma − 5.34 mm to -6.64 mm following Franklin and Paxinos mouse brain atlas [35]. The sections were preserved in an anti-freezing solution (50% PBS 0.01 M, 30% ethylene, and 20% glycerol) at -20 °C until staining. OBs were also cut at 20 μm, but directly mounted on Superfrost slides (Thermo Fisher Scientific), dried at room temperature (RT) (20–26 °C), and stored at -80 °C until staining.

### Immunostaining

Wako 019-19741 (1:1000 dilution) rabbit anti-IBA-1 (ionized calcium-binding adaptor protein-1) was used to stain for microglia and Dako Z0334 (1:1000 dilution) rabbit anti-GFAP (glial fibrillary acidic protein) to stain for astrocytes. Duplicate sections of free-floating brain slices or mounted OBs were selected within the following brain coordinates: CA1 (cornu Ammonis) and cortex (between bregma − 1.94 mm and − 2.30 mm), VN (vestibular nuclei) and cerebellum (between bregma − 5.88 mm and − 6.12 mm), and OB (between bregma 4.28 mm and 3.20 mm). The specified regions from the OB, VN, and cerebellum were selected because they present abundant neurodegeneration in *Ndufs4 KO* mice, whereas cortex and CA1 were selected as control regions with no neurodegeneration described [31, 32].

For immunostaining, sections were washed once in PBS and then blocked in 1% BSA and 0.2% Triton X-100 in PBS solution for 1 h at RT. Slides were then incubated overnight at 4 °C with the chosen primary antibody diluted in blocking solution. The following day, sections were left for 1 h at RT, washed three times with PBS (10 min each), and incubated with the specific secondary antibody diluted in blocking solution 1 h at RT carefully protected from light (1:600 goat anti-rabbit Alexa Fluor 568 Thermofisher A11011). Finally, sections were washed again three times with PBS (10 min each), and free-floating brain slices were mounted on Superfrost slides (Thermo Fisher Scientific). The slides were then dried and mounted with DAPI Fluoromount (Southern Biotech).

### Microscopy and image quantification

Immunofluorescence images were captured using a Nikon Eclipse 90i microscope coupled to a Nikon Digital Camera DXM1200F, controlled by the ACT-1 v2.70 capture software (Nikon, Tokyo, Japan). Images of the entire CA1 and VN were taken at 20X magnification, while images of the entire cortex, OB, and cerebellum slices were taken at 10X magnification. To ensure accuracy and consistency, specific detector sensitivity conditions were established for each area and the exposure and gain settings were optimized for each staining and kept constant throughout the acquisition process. In the image analysis, it is important to note that we considered the entire region without subdivision into different layers or subregions, ensuring that the analysis encompassed the entirety of the structures under examination. The quantification of samples was performed using ImageJ software (FIJI version 1.51), where fluorescence intensity was measured and normalized by area. The intensity was reported in arbitrary units (a.u.) as the output values from the ImageJ software do not have a specific measurement unit. The total number of IBA-1 + cells was manually counted and relativized by area. Samples identities during image acquisition and quantification were not blinded.

### Representation and statistics

All graphics were generated using GraphPad Prism 8 software (GraphPad Software, Inc, San Diego, CA, USA). The majority of the graphics are shown as mean ± SEM, except for tidal volume and respiratory frequency (box and whisker plot), and survival (Kaplan-Meier curve). In addition, the “N” of the groups is consistently shown within the bars or next to the graph. Statistical analyses were performed using the Statistical Package for Social Sciences (SPSS) 19 (IBM, Armonk, NY, USA). Survival was analyzed using log-rank test. For comparisons between two groups (i.e., clasping), unpaired Student’s t-test was used. When there were two variables to compare, statistical analysis was performed using the generalized linear model (GzLM) [36]. The GzLM is more flexible than two-way ANOVA since it tolerates different distributions, heterogeneity of variances, and missing values. Like the two-way ANOVA, the GzLM tests for the two main effects and the interaction between them. Only when the interaction was significant, we performed a sequential Bonferroni *post hoc* test for pairwise comparisons; relevant *p*-values are shown in brackets throughout the text. Similarly, for repeated measures (body weight), a generalized estimating equation (GEE) analysis was used. Statistical significance was defined as *p*-value ≤ 0.05. Both male and female mice were used in this study, and sex differences were reported when applicable. Changes in “N” are attributed to various factors, including damaged sections (IF), reagent limitations (multiplex), or time constraints (plethysmography, clasping). For instance, it should be noted that clasping assessments were not conducted on weekends.

## Results

In previous reports, we and others described that decreasing the neuroimmune response by depleting microglial cells has beneficial effects in *Ndufs4 KO* mice [17, 18, 37]. In this study, we wanted to assess whether a chronic induction of a neuroinflammatory state prior to neuropathology onset could affect the pathology of *Ndufs4 KO* mice. For that purpose, we took advantage of the GFAP-IL6 mouse [28]. As mentioned above, GFAP-IL6 mice overexpress IL-6 guided by the *Gfap* promoter, which confines the overexpression to astrocytes, producing early postnatal astrocyte and microglial reactivity across the brain [28, 30, 38].

### Astrocytic-targeted IL-6 overexpression reduces female but not male *Ndufs4 KO* mice survival

**Fig. 1:**
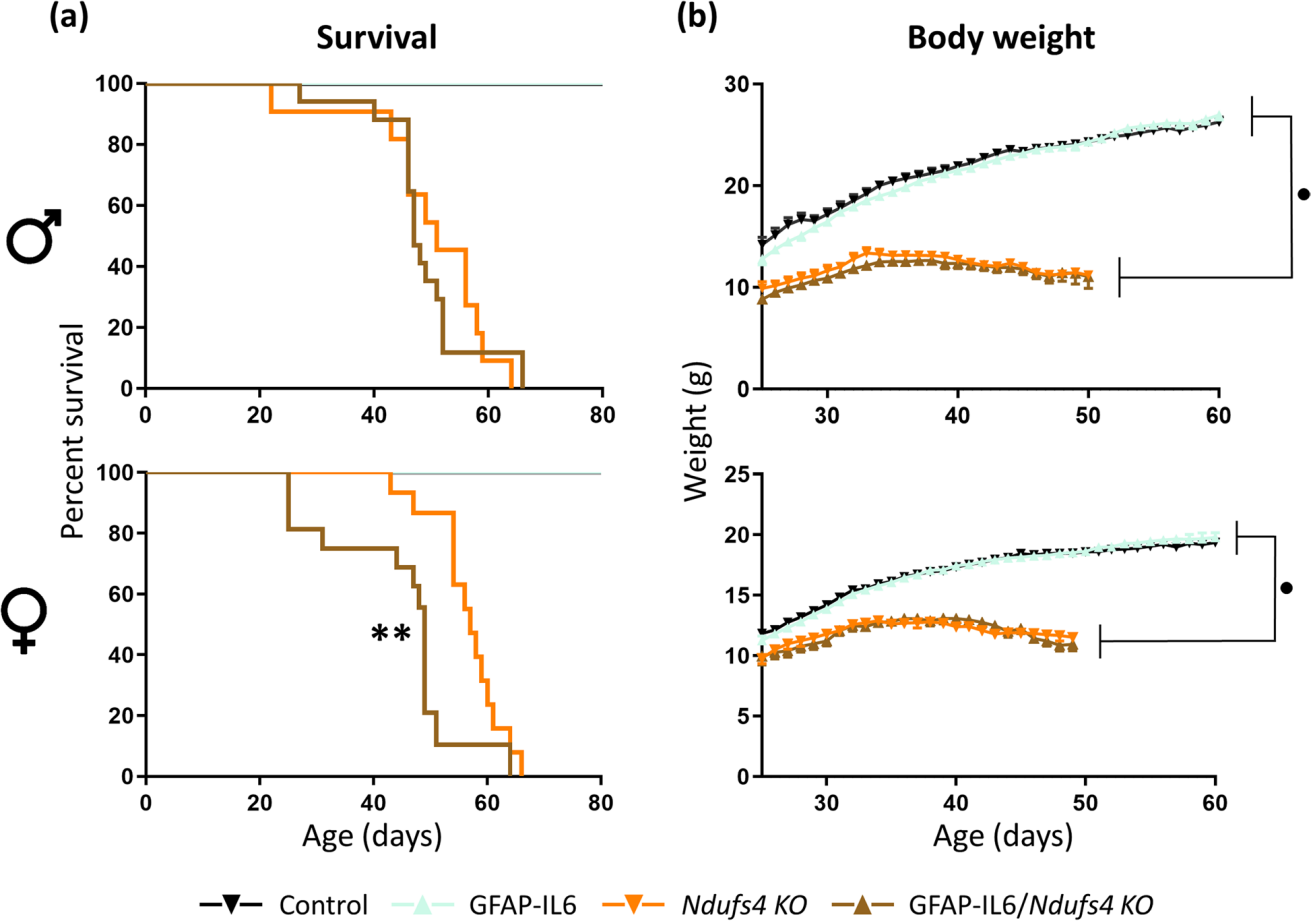
Reduced survival of female *Ndufs4 KO*mice with astrocytic IL-6 overexpression.** a** Kaplan-Meier survival curve for control (males, *n* = 17; females, *n* = 16), GFAP-IL6 (males, *n* = 17; females, *n* = 15), *Ndufs4 KO* (males, *n* = 11; females, *n* = 14), and GFAP-IL6/*Ndufs4 KO* mice (males, *n* = 17; females, *n* = 17). **b** Body weight curves for control, GFAP-IL6, *Ndufs4 KO*, and GFAP-IL6/*Ndufs4 KO* male and female mice (*n* = 11–25). ** *p* ≤ 0.01, ● *Ndufs4* effect *p* ≤ 0.05

IL-6 overexpression negatively impacted the survival of *Ndufs4 KO* mice in a sex-dependent manner: female GFAP-IL6/*Ndufs4 KO* mice showed a decreased mean survival of approximately 20% (*Ndufs4 KO* vs. GFAP-IL6/*Ndufs4 KO*, *p* = 0.0019), whereas no significant differences were found in GFAP-IL6/*Ndufs4 KO* male mice compared to *Ndufs4 KO* mice (Fig. 1a). Moreover, male and female NDUFS4-deficient mice presented the typical severe decrease in body weight, but no differences were found between*Ndufs4 KO* and GFAP-IL6/*Ndufs4 KO* mice in either sex (Fig. 1b).

### Astrocyte-targeted IL-6 overexpression barely modifies other phenotypic aspects of *Ndufs4 KO* mice

**Fig. 2:**
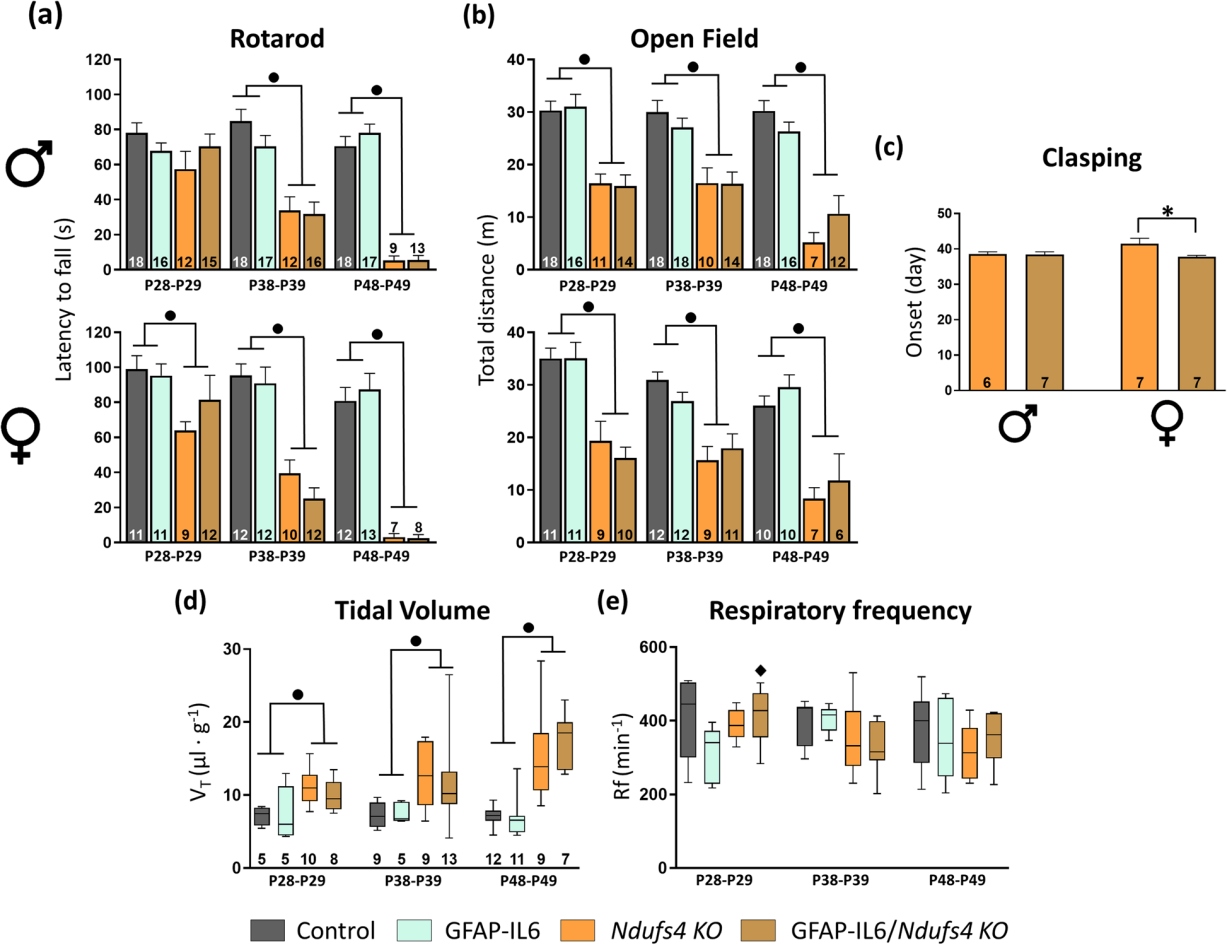
Astrocyte-tagged IL-6 overexpression barely affects the motor or respiratory phenotype of *Ndufs4 KO* mice. **a** Latency to fall in the accelerating rotarod. **b **Total distance travelled in the OF test. **c** Day of onset of clasping behavior in *Ndufs4 KO* and GFAP-IL6/*Ndufs4 KO* mice. **d** Tidal volume and (**e**) respiratory frequency at the different stages. In **d** and **e** “n” values are indicated above the X axis. ● *Ndufs4* effect *p*≤ 0.05, ★ GF-IL6 effect *p*≤ 0.05, ♦ *p*≤ 0.05 interaction between both factors, *p = 0.05–0.01

 Given that astrocytic IL-6 overexpression had a negative impact on female *Ndufs4 KO* mice survival while not affecting males, we studied the disease progression in each sex separately (Fig. 2). In general, both female and male NDUFS4-deficient mice show reduced motor coordination (Fig. 2a) and total motor activity (Fig. 2b) along with increased tidal volume (Fig. 2d), and an unchanged respiratory frequency (Fig. 2e) when compared to control mice. As for the effects of IL-6 overexpression, GFAP-IL6 mice were comparable to their controls in terms of motor coordination (Fig. 2a), motor activity (Fig. 2b), tidal volume (Fig. 2d), and respiratory frequency (Fig. 2e). In addition, IL-6 overexpression itself barely impacted the *Ndufs4 KO* phenotype regardless of sex; GFAP-IL6/*Ndufs4 KO* mice of both sexes had similar motor coordination, total motor activity, and ventilatory function as *Ndufs4 KO* mice at all disease stages (Fig. 2a, b, d, e). Only female GFAP-IL6/*Ndufs4 KO* showed a slightly advanced clasping onset (*p* = 0.023) (Fig. 2c), which is consistent with their decreased lifespan. On the contrary, we found a significant interaction between both factors in the respiratory frequency at the early stage; *post hoc* pairwise comparison revealed that NDUFS4 deficiency increased respiratory frequency in GFAP-IL6 mice (GFAP-IL6 vs. GFAP-IL6/*Ndufs4 KO*, *p* = 0.037) (Fig. 2e). Overall, these results suggest that a chronic IL-6-mediated neuroinflammatory state in *Ndufs4 KO* brains does not strongly affect disease progression.

#### NDUFS4 deficiency compromises cerebellar IL-6 production in GFAP-IL6 mice

**Fig. 3:**
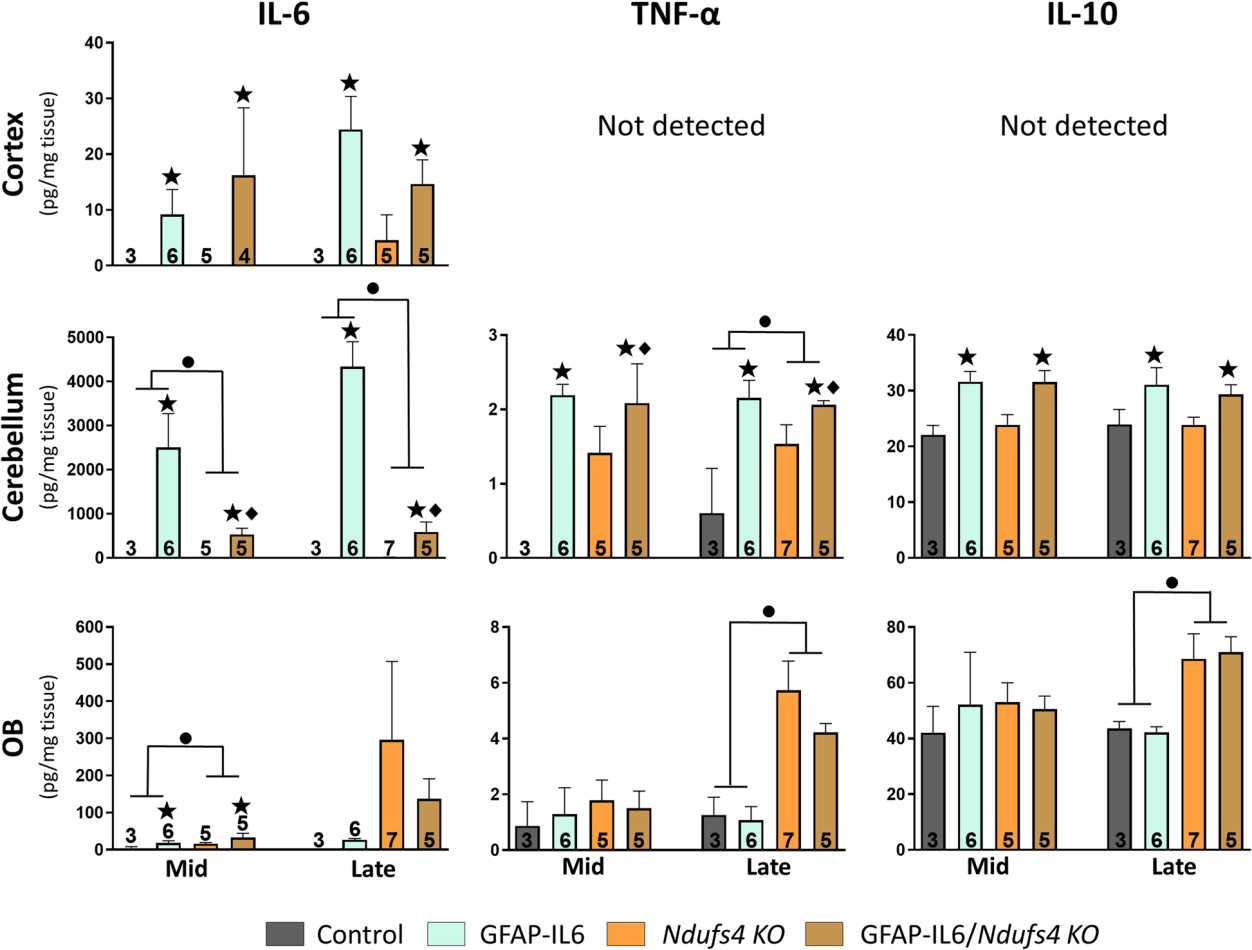
Region-dependent cytokine levels. IL-6, TNF-α, and IL-10 levels in the cortex, the cerebellum, and the olfactory bulb of the different genotypes at the mid and late stages. ● *Ndufs4* effect *p*≤ 0.05, ★ GF-IL6 effect *p*≤ 0.05, ♦ *p*≤ 0.05 interaction between both factors

 After characterizing the effects of IL-6 overexpression in *Ndufs4 KO* mice in survival and disease progression we next assessed the levels of specific cytokines at both mid (P38 – P39) and late stages (P48 – P49) in the cortex, the cerebellum, and the OB of the different genotypes (Fig. 3). As expected, IL-6 levels were increased in all regions of GFAP-IL6 mice compared to non-overexpressing mice. Noteworthy, the cerebellum had, by far, the most prominent IL-6 production in GFAP-IL6 mice, something that has been previously described [28, 39] Regarding the effects of NDUFS4 deficiency we found increased levels of IL-6 at mid and late stages in the cerebellum of NDUFS4-deficient mice. However, in the OB, the increase was only statistically significant at the mid stage as the high variability among*Ndufs4 KO* mice at the late stage precluded statistical significance (Fig. 3). Strikingly, cerebellar IL-6 levels were around five and seven times lower at mid and late stages, respectively, in GFAP-IL6/*Ndufs4 KO* mice compared to GFAP-IL6 (mid, *p* = 0.003; late, *p* < 0.001) (Fig. 3), and a similar trend in the cortex at the late stage. Regarding TNF-α levels in the OB, there were no differences at the mid stage between genotypes, while NDUFS4-deficiency increased them at the late stage. IL-6 overexpression did not have any effect at any stage (Fig. 3). In the cerebellum, both NDUFS4 deficiency and IL-6 overexpression induced an increase in TNF-α levels. After decomposing the interactions, we found increased TNF-α levels in *Ndufs4 KO* mice compared to control mice at both stages (mid, *p* = 0.017; late, *p* = 0.018), but no difference between GFAP-IL6 mice and GFAP-IL6/*Ndufs4 KO* mice (Fig. 3). IL-10, a cytokine with anti-inflammatory functions, was increased by IL-6 overexpression but not by NDUFS4 deficiency in the cerebellum at both stages. On the contrary, in the OB, IL-10 levels were increased by NDUFS4 deficiency only at the late stage, but not by IL-6 overexpression (Fig. 3). Moreover, we were unable to detect measurable levels of TNF-α and IL-10 in the cortex (Fig. 3), as well as IL-1β (not shown) in any of the regions.

### NDUFS4 deficiency decreases microgliosis in a region-dependent manner in response to chronic IL-6 overexpression at both mid and late stages of the disease

We next studied the glial reactivity by IBA-1 (microglia) and GFAP (astrocytes) staining at both mid and late stages of the disease. Importantly, we did not detect any sex-specific differences in gliosis, so the results of both sexes are presented together (Supplementary Figs. 1 and 2).

**Fig. 4:**
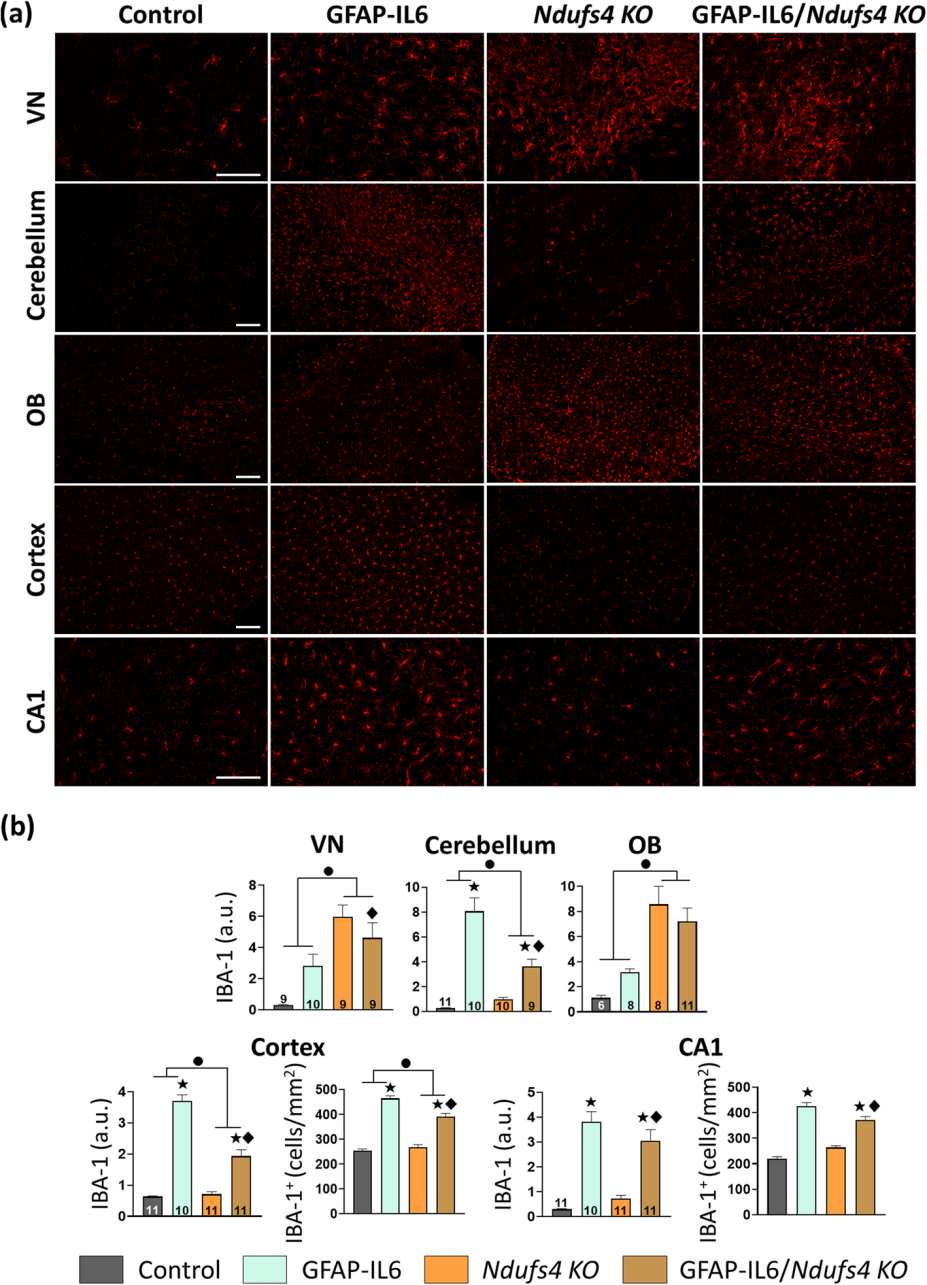
Analysis of microgliosis in the late stage mice in different brain regions revealed a region-dependent decrease in microgliosis in GFAP-IL6 mice due to NDUFS4 deficiency.** a** Representative images of IBA-1 staining. Scale bar 100 μm. **b** Quantification of IBA-1 fluorescence intensity in the VN, cerebellum, OB, cortex, and CA1, as well as the number of IBA-1^+^ cells in the cortex and CA1 of the different genotypes. ● *Ndufs4* effect *p* ≤ 0.05, ★ GF-IL6 effect *p* ≤ 0.05, ♦ *p* ≤ 0.05 interaction between both factors

As expected, at the late stage (Fig. 4), NDUFS4 deficiency led to an increased IBA-1 fluorescence intensity in the VN, the OB, and the cerebellum of *Ndufs4 KO* mice, indicating a significant presence of reactive microglia, especially in the VN and the OB. However, in the CA1 and the cortex, both IBA-1 intensity and the number of IBA-1^+^ cells were comparable to control mice (Fig. 4). Regarding IL-6 overexpression effects, we found a significant interaction between both factors in the VN. This interaction indicated that IL-6 overexpression had no major impact on the IBA-1 intensity of *Ndufs4 KO* mice (*Ndufs4 KO* vs. GFAP-IL6/*Ndufs4 KO*, *p* = 0.172). However, IL-6 overexpression induced a clear increase in NDUFS4 non-deficient mice (Control vs. GFAP-IL6, *p* = 0.027), probably because the massive microglia reactivity of NDUFS4-deficient mice in this region is mainly due to the effect of the genotype without the influence of IL-6 (Fig. 4a). Accordingly, GFAP-IL6/*Ndufs4 KO* mice developed the characteristic bilateral symmetrical lesions observed in *Ndufs4 KO* mice, with no apparent differences between genotypes (Fig. 4b). In the other regions, IL-6 overexpression clearly induced a substantial increase in IBA-1 fluorescence intensity in both genotypes. Interestingly, even though *Ndufs4 KO* mice responded to IL-6 overexpression by increasing both IBA-1 intensity and the number of IBA-1^+^ cells, they did it to a lesser extent when compared with GFAP-IL6 mice in the cerebellum, cortex, and CA1 (GFAP-IL6 vs. GFAP-IL6/*Ndufs4 KO*, *p* ≤ 0.001 in all regions) (Fig. 4).

**Fig. 5:**
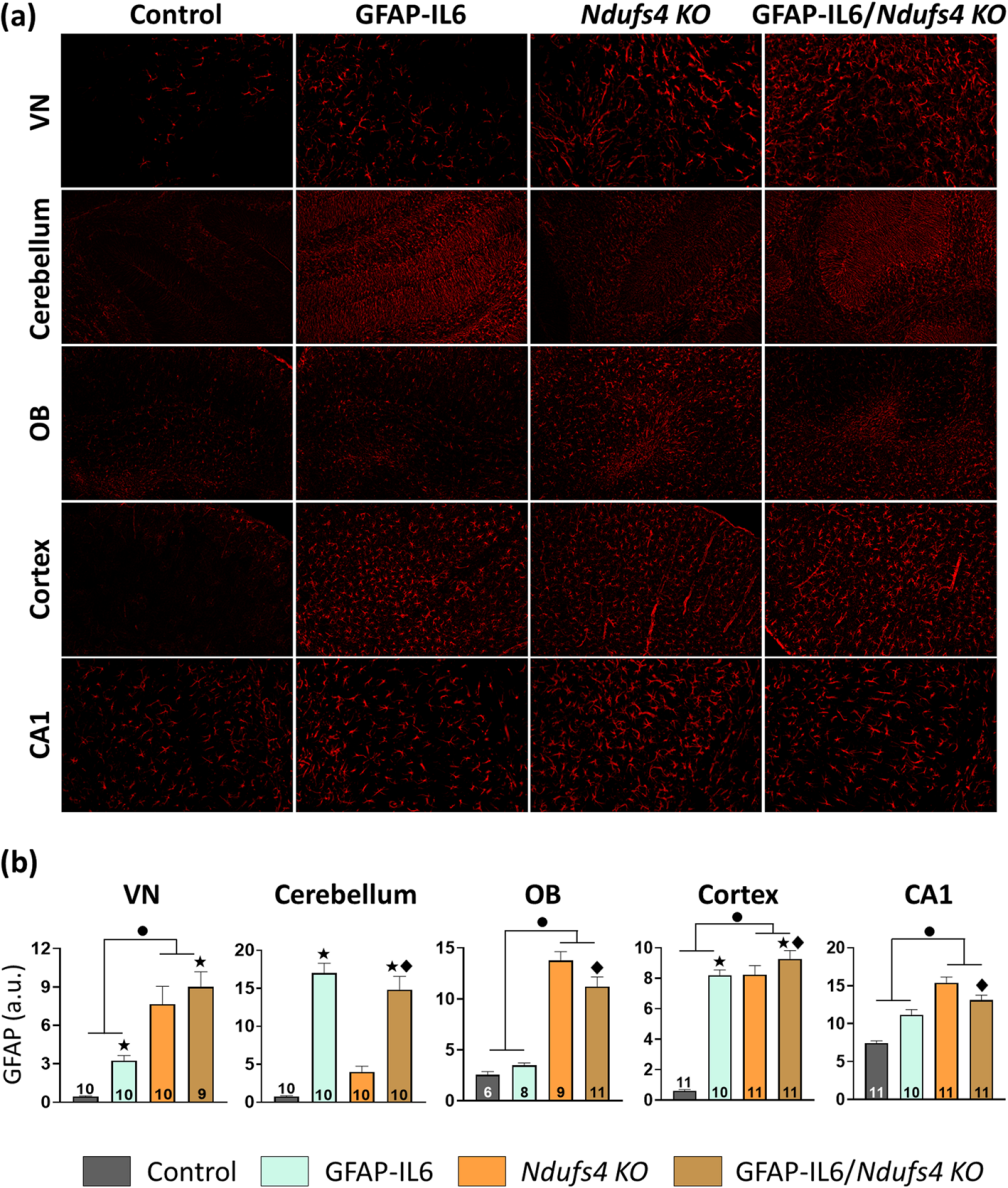
IL-6 overexpression effects over GFAP are region-dependent in *Ndufs4KO* mice at the late stage.** a** Representative images of GFAP staining. Scale bar 100 μm. **b** GFAP mean fluorescence intensity quantification in VN, cerebellum, OB, cortex, and CA1. ● *Ndufs4* effect *p* ≤ 0.05, ★ GF-IL6 effect *p* ≤ 0.05, ♦ *p* ≤ 0.05 interaction between both factors

Consistent with microgliosis and in agreement with previous reports [17, 32, 40], we found a widespread increase of GFAP in late stage *Ndufs4 KO* mice in the VN, cerebellum, OB, cortex, and CA1 (Fig. 5). IL-6 overexpression also induced a clear increase in GFAP in GFAP-IL6 mice compared to control mice. However, in *Ndufs4 KO* mice, the effects of IL-6 overexpression varied depending on the brain region. In the VN and the cerebellum, IL-6 overexpression further increased GFAP fluorescence intensity in *Ndufs4 KO* mice (IL-6 effect, *p* = 0.013 and *p* ≤ 0.001, respectively). On the other hand, it had no effect in the cortex (*Ndufs4 KO* vs. GFAP-IL6/*Ndufs4 KO*, *p* = 0.233), and reduced GFAP intensity in the OB and the CA1 (*Ndufs4 KO* vs. GFAP-IL6/*Ndufs4 KO*, *p* = 0.026 and p = 0.009, respectively) (Fig. 5b).

**Fig. 6:**
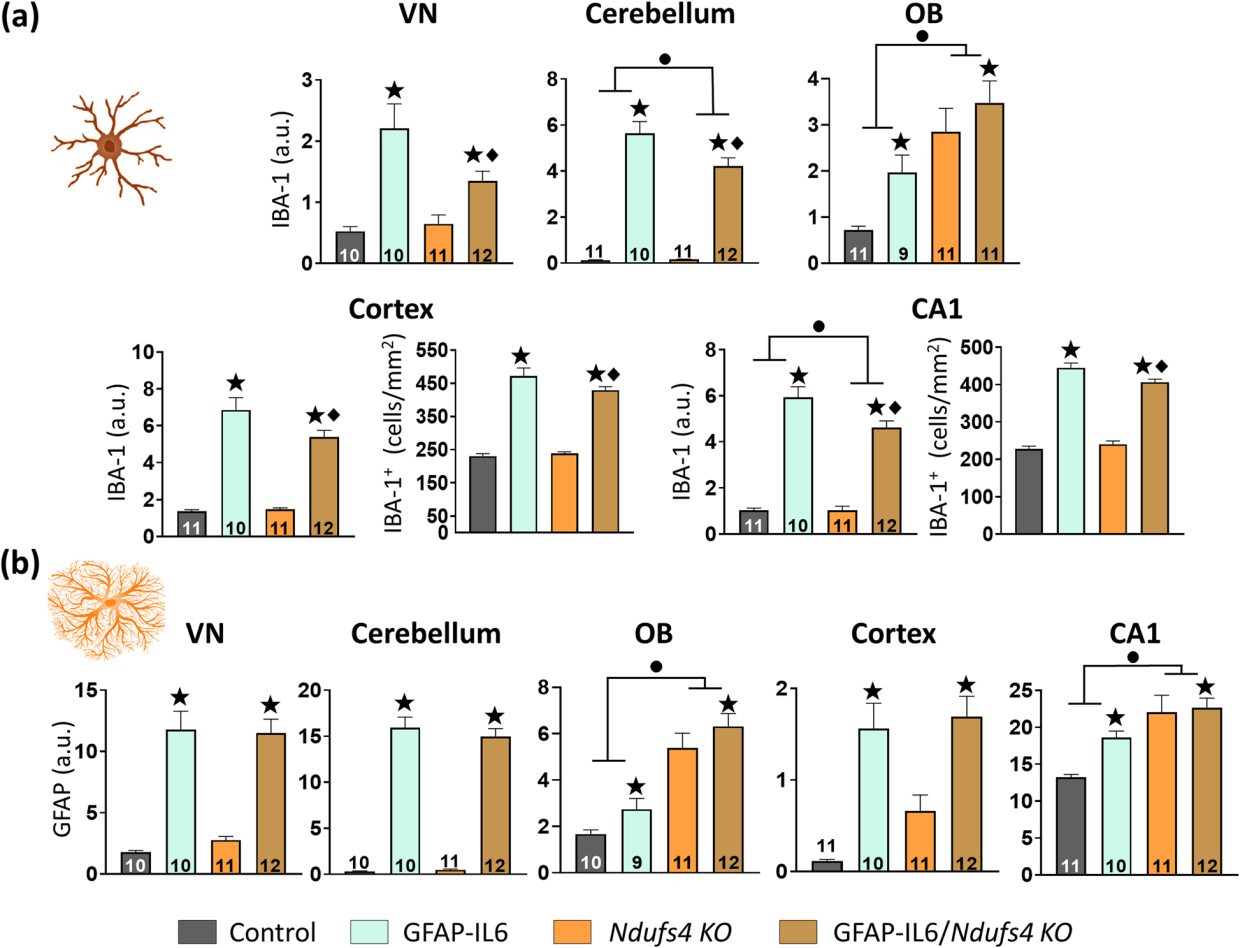
Microgliosis, but not astrogliosis, is decreased in a region-dependent manner in GFAP-IL6 mice due to NDUFS4 deficiency at the mid stage.** a** Quantification of IBA-1 fluorescence intensity in the VN, cerebellum, OB, cortex, and CA1, as well as the number of IBA-1^+^ cells in the cortex, and CA1 of the different genotypes. **b** GFAP mean fluorescence intensity quantification in the VN, cerebellum, OB, cortex, and CA1. ● *Ndufs4* effect *p* ≤ 0.05, ★ GF-IL6 effect *p* ≤ 0.05, ♦ *p* ≤ 0.05 interaction between both factors

At the mid stage (Fig. 6), we only found overt signs of microgliosis in the OB of *Ndufs4 KO* mice, but not in any other region (Fig. 6a). IL-6 overexpression again induced a clear increase in IBA-1 in all regions, notwithstanding the genotype. In line with the late stage observations, we again found a decrease in IBA-1 in GFAP-IL6/*Ndufs4 KO* mice when compared to GFAP-IL6 mice in the cerebellum, cortex, and CA1. We also observed a decrease in the VN (GFAP-IL6 vs. GFAP-IL6/*Ndufs4 KO*, *p* = 0.015), probably because, contrary to the late stage, microglial reactivity in response to NDUFS4 deficiency in the VN is incipient and does not mask the effect of IL-6 overexpression. Remarkably, the magnitude of the decreasing effect appeared to be less pronounced at the mid stage, in both the fluorescence intensity and the number of cells in the CA1 and cortex when compared to the late stage (Figs. 4b and 6a).

Regarding astrocyte reactivity, we found increased GFAP intensity in the OB and the CA1 due to NDUFS4 deficiency, but not in the VN, cerebellum, or cortex. Moreover, we found increased GFAP in IL-6 overexpressing mice across all regions, regardless of the genotype. Unlike IBA-1, GFAP intensity was similar between GFAP-IL6 and GFAP-IL6/*Ndufs4 KO* mice in the VN, cerebellum, and cortex, while it was increased in the OB and the CA1 due to NDUFS4 deficiency (Fig. 6b).

## Discussion

Our main goal was to assess whether an increased inflammatory environment could influence disease progression in *Ndufs4 KO* mice. This was motivated by clinical evidence indicating that immune-associated processes could trigger the onset of MDs and also modify the pathology to some extent [21]. To do so, we used GFAP-IL6 and *Ndufs4 KO* mice to generate GFAP-IL6/*Ndufs4 KO* mice. GFAP-IL6 mice have been widely used as a model for chronic neuroinflammation [29]. As mentioned above, these mice overexpress the cytokine IL-6 under the control of the *Gfap* promoter leading to abundant microgliosis and astrogliosis, wherein both cell types show morphological signs of reactivity. IL-6 overexpression also promotes tissue vacuolization, angiogenesis, peripheral cell infiltration, and ultimately neurodegeneration [28, 29, 41]. As result, these mice start to develop serious motor problems around six months of age [42]. Consistent with this, in young mice, we found prominent microgliosis and astrogliosis but no differences in total ambulation and motor coordination between control and GFAP-IL6 mice at any time point. We first hypothesized that IL-6 overexpression would potentiate and advance disease progression in *Ndufs4 KO* mice based on extensive literature indicating that a neuroinflammatory state can be neurotoxic by aggravating neurodegenerative mechanisms [43]. Additionally, the presence of inflammatory by-products could further harm neurons that are already vulnerable to NDUFS4 deficiency [44–46]. Strikingly, IL-6 overexpression only reduced the survival and slightly advanced the onset of clasping in *Ndufs4 KO* female mice, which may indicate that *Ndufs4 KO* female mice are more vulnerable to brain inflammation than male mice, highlighting the importance of considering each sex separately in these types of studies. Apart from the differences observed in survival and clasping onset, female GFAP-IL6/*Ndufs4 KO* mice were phenotypically similar to males in terms of disease progression and gliosis. Given the severe fast-progressing disease that *Ndufs4 KO* develop, it is worth considering that specific assessments, like the rotarod test used to evaluate motor decline, might have encountered ceiling or floor effects potentially hampering our ability to detect additional changes in the motor function of GFAP-IL6/*Ndufs4 KO* mice when compared to their *Ndufs4 KO* counterparts. It cannot be ruled out that other motor-related tasks could be more sensitive to detecting further functional deterioration in GFAP-IL6/*Ndufs4 KO* mice during the progression of disease, which deserves further attention.

We did not directly interrogate the specific cause of the sex-dependent differences observed in survival, but since both GFAP-IL6/*Ndufs4 KO* female and male mice had similar disease progression compared to their *Ndufs4 KO* mice counterparts, it is unlikely that this observation is related to increased neuronal loss in females. We suggest that it may be due to alterations in other aspects of the *Ndufs4 KO* phenotype, which were not directly addressed in this study. The appearance of seizures in *Ndufs4 KO* mice have been previously observed [31, 32, 44], and there is a significant correlation between inflammation and epilepsy, as seizures can trigger inflammation or inflammation can exacerbate the severity of seizures [47]. In addition, some studies have observed different sex susceptibilities to the development of specific epilepsy subtypes [48, 49]. Therefore, it cannot be ruled out that the sex-dependent effect on survival in GFAP-IL6/*Ndufs4 KO* mice is due to a greater susceptibility to developing epilepsy, or a greater severity of seizures in GFAP-IL6/*Ndufs4 KO* female mice compared to GFAP-IL6/*Ndufs4 KO* male mice and *Ndufs4 KO* mice. Increased susceptibility to epilepsy in females is consistent with the lack of a significant decrease in body weight before death in female mice. In summary, despite this decreased survival in GFAP-IL6/*Ndufs4 KO* female mice compared to *Ndufs4 KO* mice, IL-6 overexpression had only a minor impact on the disease progression indicating that chronically boosting inflammation by overexpressing IL-6 in the brain of *Ndufs4 KO* mice is not sufficient to accelerate disease.

Surprisingly, NDUFS4 deficiency partially compromised cerebellar IL-6 production in GFAP-IL6 mice. GFAP levels measured by immunostaining were comparable between both IL-6 overexpressing mice in the cerebellum and other regions at both disease stages. Since *Il6* expression is guided by the *Gfap* promoter in GFAP-IL6 mice, one might anticipate a correlation between GFAP and IL-6 protein levels given their mRNA-coupled expression. However, it is well known that mRNA and protein levels do not always correlate [50, 51]. Additionally, the levels of GFAP immunoreactivity were already strongly elevated in GFAP-IL6 mice, suggesting that GFAP protein levels may be approaching saturation, even though *Gfap* transcription may still be active, and thus *Il6*. No studies have described the dynamics between *Gfap* and *Il6* gene and protein expression in the context of GFAP-IL6 mice. Indeed, only a few studies have quantified IL-6 protein levels in GFAP-IL6 mice so far [39, 52]. On this matter, our results evidence that GFAP immunoreactivity and IL-6 levels do not necessarily correlate in GFAP-IL6 mice. We have formulated two hypotheses to explain why IL-6 is consistently decreased at both mid and late stages in the cerebellum of GFAP-IL6/*Ndufs4 KO* mice when compared to GFAP-IL6 mice. The first explanation is that NDUFS4 deficiency directly affects astrocytic IL-6 production in this region. It is possible that OXPHOS defective astrocytes could not entirely sustain such an abundant IL-6 production in the cerebellum [53]. Another possibility is that NDUFS4-deficient microglia are not able to respond to an inflammatory stimulus such as IL-6 appropriately. In the normal scenario (GFAP-IL6 mice) and given that microglia are important target and effector cells of this cytokine, it was assumed that the primary IL-6 released by astrocytes would engage a paracrine immune response in microglia, which then would release other inflammatory mediators. These microglia-released factors could further exacerbate inflammation, leading to increased astrocyte reactivity and upregulated expression of *Gfap*, which, in turn, promotes the production of IL-6, establishing a positive feedback loop. However, a very recent work seems to contradict this previous assumption; the authors found that microglia-depleted GFAP-IL6 mice had similar *Il6* expression levels and comparable levels of astrocyte reactivity measured by GFAP [54], indicating that, in principle, astrocytes could produce identical amounts of IL-6 and become similarly reactive without any microglial input in GFAP-IL6 mice.

Furthermore, we consistently observed a decreased microgliosis in response to IL-6 overexpression in *Ndufs4 KO* mice across several regions and at both time points studied. Our main hypothesis is that this is due to an abnormal microglial response in NDUFS4-deficient mice to stimulation with IL-6, rather than to the levels of IL-6 alone. This conclusion is supported by the fact that, this decrease in microgliosis was consistently observed in both the cerebellum and cortex, where IL-6 levels were decreased and unchanged, respectively, between GFAP-IL6/*Ndufs4 KO* and GFAP-IL6 mice. To our knowledge, the great majority of studies that have investigated the metabolic and mitochondrial adaptations of microglia in response to an inflammatory stimulus have used lipopolysaccharide (LPS). LPS is a component of the cell wall of gram-negative bacteria that stimulates microglia via Toll-like receptor 4 (TLR4), leading to the production of pro-inflammatory cytokines and the activation of the innate immune response [55]. These studies found that LPS stimulation switches the microglial metabolic profile from OXPHOS to glycolysis (a process known as glycolytic switch) in rodents. This switch to glycolysis is necessary for the production of pro-inflammatory cytokines, highlighting the crucial role of metabolic reprogramming in the immune response to LPS and potentially to other pro-inflammatory stimuli [56–59]. Interestingly, LPS stimulation has also been reported to induce a transient increase in microglial OXPHOS O_2_ consumption during this conversion to glycolysis [60]. Additionally, other mitochondrial processes, including mitochondrial dynamics, have been implicated in the microglial response to LPS [60–63]. It is then possible that microglia could exhibit different responses to IL-6 stimulation due to a compromised complex I function.

## Conclusions

Neuroinflammation is a common hallmark of primary mitochondrial diseases and neurodegenerative processes, having been proposed as a trigger of neuronal death and a key mediator in the amplification of pathological processes. Here, we provide evidence that, based on the measurements we have employed, chronic neuroinflammation induced by continuous IL-6 overexpression does not substantially impact the phenotype of a well-established Leigh Syndrome model. On the contrary, our results underscore the contribution of mitochondrial dysfunction to microglial reactivity, providing novel evidence of the necessity of intact mitochondrial function for microglial responses.

### Supplementary Information

** Additional file 1:** **Supplementary Figure 1.** Microgliosis and astrogliosis at the late stage divided by sex. Related with Figures 4 and 5. (**a**) Quantification of IBA-1 fluorescence intensity in the VN, cerebellum, OB, cortex, and CA1, as well as the number of IBA-1^+^ cells in the cortex, and CA1 of the different genotypes. (**b**)GFAP mean fluorescence intensity quantification in the VN, cerebellum, OB, cortex, and CA1. ● *Ndufs4 *effect *p*≤ 0.05,  ★ GF-IL6 effect *p*≤ 0.05, ♦ *p*≤ 0.05 interaction between both factors. **Supplementary Figure 2.** Microgliosis and astrogliosis at the mid stage divided by sex. Related with Figure 6. (a) Quantification of IBA-1 fluorescence intensity in the VN, cerebellum, OB, cortex, and CA1, as well as the number of IBA-1^+^ cells in the cortex, and CA1 of the different genotypes. (**b**) GFAP mean fluorescence intensity quantification in the VN, cerebellum, OB, cortex, and CA1. ● *Ndufs4 *effect *p*≤ 0.05,  ★ GF-IL6 effect *p*≤ 0.05, ♦ *p*≤ 0.05 interaction between both factors.

** Additional file 2:** Supplementary tables with the statistical outputs of the GEE or GzLM analyses corresponding to the results of the main text.
