# Supplementary material for: Interleukin-6-elicited chronic neuroinflammation may decrease survival but is not sufficient to drive disease progression in a mouse model of Leigh syndrome
Source: J Inflamm (Lond). 2024 Jan 11;21:1. doi: 10.1186/s12950-023-00369-4 (PMC10782699; doi:10.1186/s12950-023-00369-4)
Supplement: Supplementary file 1 — Additional file 1: Supplementary Figure 1. Microgliosis and astrogliosis at the late stage divided by sex. Related with Figures 4 and 5. (a) Quantification of IBA-1 fluorescence intensity in the VN, cerebellum, OB, cortex, and CA1, as well as the number of IBA-1+ cells in the cortex, and CA1 of the different genotypes. (b)GFAP mean fluorescence intensity quantification in the VN, cerebellum, OB, cortex, and CA1. ● Ndufs4 effect p≤ 0.05, ★ GF-IL6 effect p≤ 0.05, ♦ p≤ 0.05 interaction between both factors. Supplementary Figure 2. Microgliosis and astrogliosis at the mid stage divided by sex. Related with Figure 6. (a) Quantification of IBA-1 fluorescence intensity in the VN, cerebellum, OB, cortex, and CA1, as well as the number of IBA-1+ cells in the cortex, and CA1 of the different genotypes. (b) GFAP mean fluorescence intensity quantification in the VN, cerebellum, OB, cortex, and CA1. ● Ndufs4 effect p≤ 0.05, ★ GF-IL6 effect p≤ 0.05, ♦ p≤ 0.05 interaction between both factors. [file 12950_2023_369_MOESM1_ESM.pptx]

## Slide 1
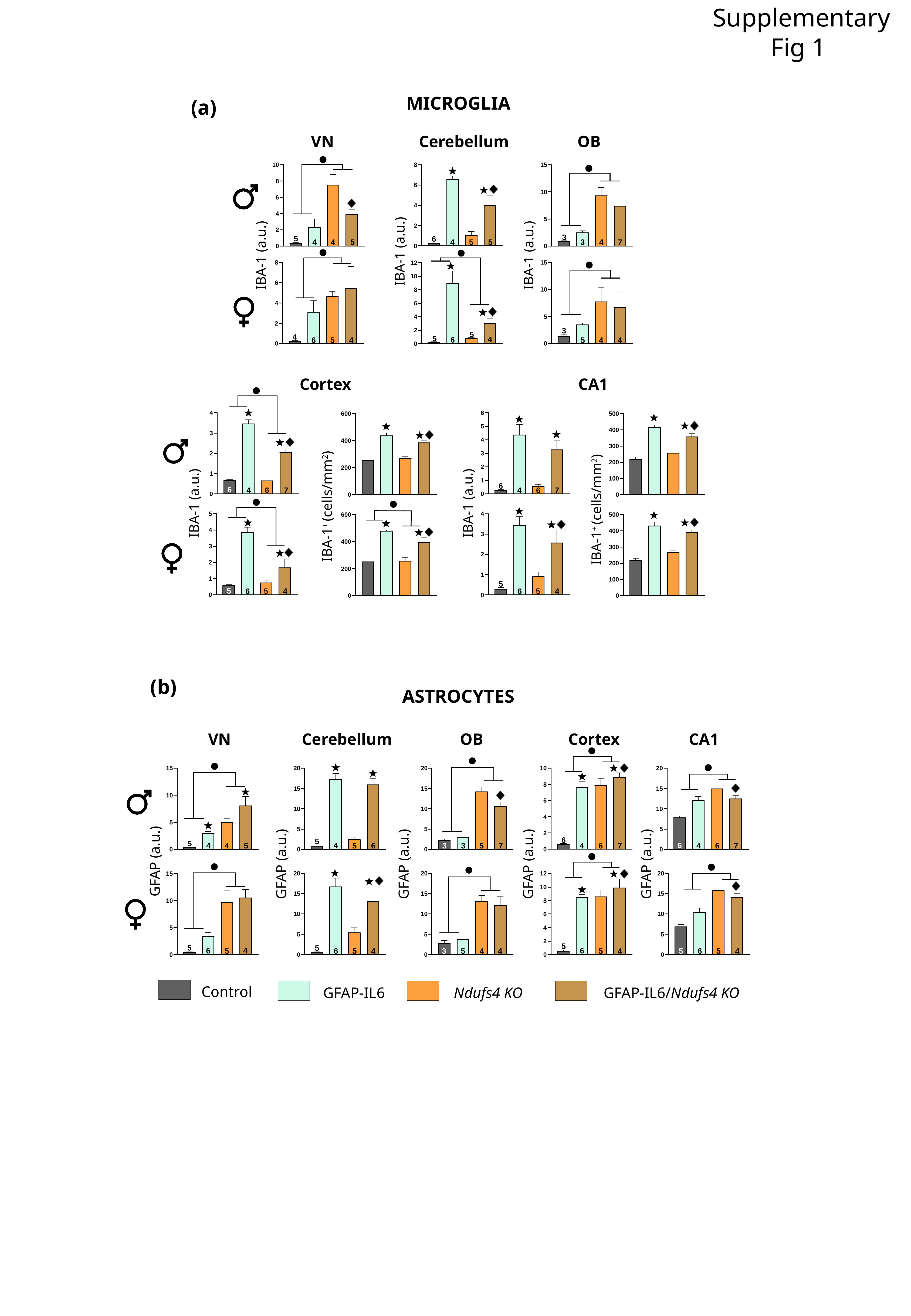

Supplementary Fig 1
MICROGLIA
(a)
VN
Cerebellum
OB
IBA-1 (a.u.)
IBA-1 (a.u.)
IBA-1 (a.u.)
Cortex
CA1
IBA-1+ (cells/mm2)
IBA-1+ (cells/mm2)
IBA-1 (a.u.)
IBA-1 (a.u.)
(b)
ASTROCYTES
VN
Cerebellum
OB
Cortex
CA1
GFAP (a.u.)
GFAP (a.u.)
GFAP (a.u.)
GFAP (a.u.)
GFAP (a.u.)
Control
GFAP-IL6
Ndufs4 KO
GFAP-IL6/Ndufs4 KO

## Slide 2
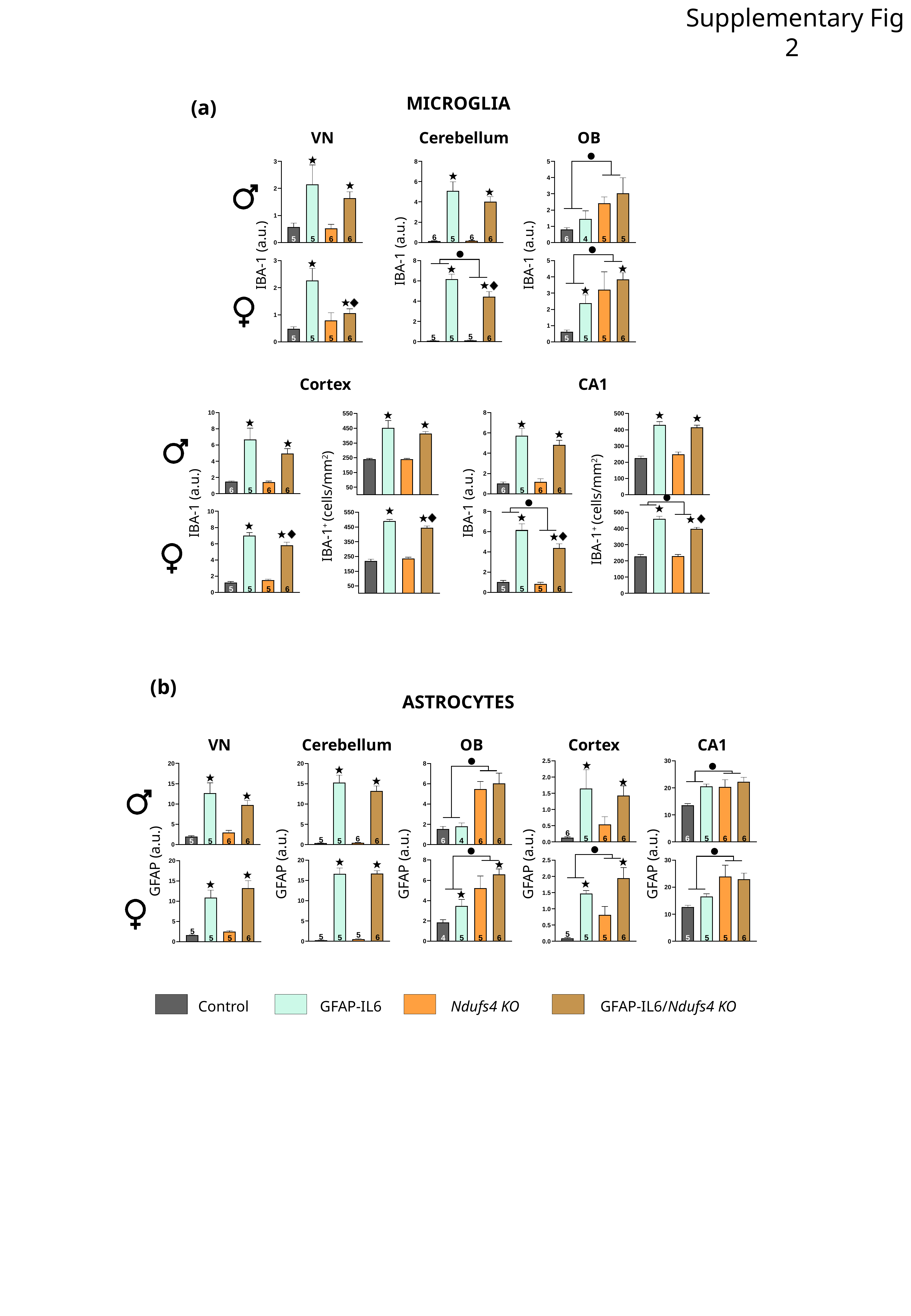

Supplementary Fig 2
MICROGLIA
(a)
VN
Cerebellum
OB
IBA-1 (a.u.)
IBA-1 (a.u.)
IBA-1 (a.u.)
Cortex
CA1
IBA-1+ (cells/mm2)
IBA-1+ (cells/mm2)
IBA-1 (a.u.)
IBA-1 (a.u.)
(b)
ASTROCYTES
VN
Cerebellum
OB
Cortex
CA1
GFAP (a.u.)
GFAP (a.u.)
GFAP (a.u.)
GFAP (a.u.)
GFAP (a.u.)
Control
GFAP-IL6
Ndufs4 KO
GFAP-IL6/Ndufs4 KO
