# Supplementary material for: Interleukin-6-elicited chronic neuroinflammation may decrease survival but is not sufficient to drive disease progression in a mouse model of Leigh syndrome
Source: J Inflamm (Lond). 2024 Jan 11;21:1. doi: 10.1186/s12950-023-00369-4 (PMC10782699; doi:10.1186/s12950-023-00369-4)
Supplement: Supplementary file 2 — Additional file 2: Supplementary tables with the statistical outputs of the GEE or GzLM analyses corresponding to the results of the main text. [file 12950_2023_369_MOESM2_ESM.docx]

Tables with the outputs of the GEE or GzLM statistical analysis. Ndufs4 represents the NDUFS4 deficiency effect, GF-IL6 represents the IL-6 overexpression effect, and Ndufs4*GF-IL6 represent the interaction between both factors. Statistical significant results are highlighted in bold (p ≤ 0.05). χ^2^ , Wald chi-square; degrees of freedom, df; p, p-value.

**Figure 1**

| Body weight  (Figure 1b) | Males | **Ndufs4: χ^2^ = 753.05, df (1), p ≤ 0.001**  GF-IL6: χ^2^ = 1.56, df (1), p = 0.211  Ndufs4*GF-IL6: χ^2^ = 0.001, df (1), p = 0.976 |
| --- | --- | --- |
|  | Females | **Ndufs4: χ^2^ = 414.37, df (1), p ≤ 0.001**  GF-IL6: χ^2^ = 0.014, df (1), p = 0.904  Ndufs4*GF-IL6: χ^2^ = 0.7 , df (1), p = 0.792 |

**Figure 2**

| Rotarod  (Figure 2a) | Males | Early | Ndufs4: χ^2^ = 1.907, df (1), p = 0.167  GF-IL6: χ^2^ = 0.045 , df (1), p = 0.832  Ndufs4*GF-IL6: χ^2^ = 3.13 , df (1), p = 0.077 |
| --- | --- | --- | --- |
|  |  | Mid | **Ndufs4: χ^2^ = 44.96, df (1), p ≤ 0.001**  GF-IL6: χ^2^ = 1,51 , df (1), p = 0.219  Ndufs4*GF-IL6: χ^2^ = 0.865 , df (1), p = 0.352 |
|  |  | Late | **Ndufs4: χ^2^ = 205.89, df (1), p ≤ 0.001**  GF-IL6: χ^2^ = 0.672 , df (1), p = 0.412  Ndufs4*GF-IL6: χ^2^ = 0.578 , df (1), p = 0.447 |
|  | Females | Early | **Ndufs4: χ^2^ = 6.97, df (1), p = 0.008**  GF-IL6: χ^2^ = 0.55 , df (1), p = 0.458  Ndufs4*GF-IL6: χ^2^ = 1.32, df (1), p = 0.251 |
|  |  | Mid | **Ndufs4: χ^2^ = 71.25, df (1), p ≤ 0.001**  GF-IL6: χ^2^ = 1,71 , df (1), p = 0.191  Ndufs4*GF-IL6: χ^2^ = 0.459 , df (1), p = 0.498 |
|  |  | Late | **Ndufs4: χ^2^ = 117,61, df (1), p ≤ 0.001**  GF-IL6: χ^2^ = 0.159 , df (1), p = 0.69  Ndufs4*GF-IL6: χ^2^ = 0.231, df (1), p = 0.631 |
| Open Field  (Figure 2b) | Males | Early | **Ndufs4: χ^2^ = 55.13, df (1), p ≤ 0.001**  GF-IL6: χ^2^ = 0.004 , df (1), p = 0.951  Ndufs4*GF-IL6: χ^2^ = 0.101, df (1), p = 0.751 |
|  |  | Mid | **Ndufs4: χ^2^ = 29.8, df (1), p ≤ 0.001**  GF-IL6: χ^2^ = 0.491, df (1), p = 0.484  Ndufs4*GF-IL6: χ^2^ = 0.41 , df (1), p = 0.522 |
|  |  | Late | **Ndufs4: χ^2^ = 68,84, df (1), p ≤ 0.001**  GF-IL6: χ^2^ = 0.098, df (1), p = 0.754  Ndufs4*GF-IL6: χ^2^ = 3,67 , df (1), p = 0.055 |
|  | Females | Early | **Ndufs4: χ^2^ = 50.1, df (1), p ≤ 0.001**  GF-IL6: χ^2^ = 0.725, df (1), p = 0.394  Ndufs4*GF-IL6: χ^2^ = 0.733 , df (1), p = 0.392 |
|  |  | Mid | **Ndufs4: χ^2^ = 35.04, df (1), p ≤ 0.001**  GF-IL6: χ^2^ = 0.173, df (1), p = 0.678  Ndufs4*GF-IL6: χ^2^ = 2.39 , df (1), p = 0.122 |
|  |  | Late | **Ndufs4: χ^2^ = 46.02, df (1), p ≤ 0.001**  GF-IL6: χ^2^ = 1.74, df (1), p = 0.187  Ndufs4*GF-IL6: χ^2^ = 0.000 , df (1), p = 0.992 |
| Tidal Volume  (Figure 2d) | Both sexes | Early | **Ndufs4: χ^2^ = 12.05, df (1), p = 0.001**  GF-IL6: χ^2^ = 0.04, df (1), p = 0.842  Ndufs4*GF-IL6: χ^2^ = 0.03 , df (1), p = 0.864 |
|  |  | Mid | **Ndufs4: χ^2^ = 18.63, df (1), p ≤ 0.001**  GF-IL6: χ^2^ = 0.316, df (1), p = 0.574  Ndufs4*GF-IL6: χ^2^ = 0.7 , df (1), p = 0.401 |
|  |  | Late | **Ndufs4: χ^2^ = 57.48, df (1), p ≤ 0.001**  GF-IL6: χ^2^ = 0.06, df (1), p = 0.807  Ndufs4*GF-IL6: χ^2^ = 0.333, df (1), p = 0.564 |
| Respiratory frequency  (Figure 2e) | Both sexes | Early | Ndufs4: χ^2^ = 2.53, df (1), p = 0.112  GF-IL6: χ^2^ = 2.3, df (1), p = 0.13  **Ndufs4*GF-IL6: χ^2^ = 5.24 , df (1), p = 0.022** |
|  |  | Mid | Ndufs4: χ^2^ = 2.1, df (1), p = 0.147  GF-IL6: χ^2^ = 0.943, df (1), p = 0.332  Ndufs4*GF-IL6: χ^2^ = 0.846, df (1), p = 0.358 |
|  |  | Late | Ndufs4: χ^2^ = 1.85, df (1), p = 0.174  GF-IL6: χ^2^ = 0.102, df (1), p = 0.75  Ndufs4*GF-IL6: χ^2^ = 0.746, df (1), p = 0.388 |

**Figure 3**

| Cortex | IL-6 | Mid | Ndufs4: χ^2^ = 0.398, df (1), p = 0.528  **GF-IL6: χ^2^ = 5,18, df (1), p = 0.023**  Ndufs4*GF-IL6: χ^2^ = 0.398, df (1), p = 0.528 |
| --- | --- | --- | --- |
|  |  | Late | Ndufs4: χ^2^ = 0.312, df (1), p = 0.577  **GF-IL6: χ^2^ = 13.54, df (1), p ≤ 0.001**  Ndufs4*GF-IL6: χ^2^ = 2.33 , df (1), p = 0.126 |
| Cerebellum | IL-6 | Mid | **Ndufs4: χ^2^ = 4.53, df (1), p = 0.033**  **GF-IL6: χ^2^ = 10.71, df (1), p = 0.001**  **Ndufs4*GF-IL6: χ^2^ = 4.58, df (1), p = 0.032** |
|  |  | Late | **Ndufs4: χ^2^ = 32.41, df (1), p ≤ 0.001**  **GF-IL6: χ^2^ = 55.72, df (1), p ≤ 0.001**  **Ndufs4*GF-IL6: χ^2^ = 32.73, df (1), p ≤ 0.001** |
|  | TNF-α | Mid | **Ndufs4: χ^2^ = 4.12, df (1), p = 0.042**  **GF-IL6: χ^2^ = 19.66, df (1), p ≤ 0.001 Ndufs4*GF-IL6: χ^2^ = 5.6, df (1), p = 0.018** |
|  |  | Late | Ndufs4: χ^2^ = 2.54, df (1), p = 0.111  **GF-IL6: χ^2^ = 15.9, df (1), p ≤ 0.001**  **Ndufs4*GF-IL6: χ^2^ = 3.88, df (1), p = 0.049** |
|  | IL-10 | Mid | Ndufs4: χ^2^ = 0.29, df (1), p = 0.591  **GF-IL6: χ^2^ = 23,09, df (1), p ≤ 0.001**  Ndufs4*GF-IL6: χ^2^ = 0.331, df (1), p = 0.565 |
|  |  | Late | Ndufs4: χ^2^ = 0.195, df (1), p = 0.659  **GF-IL6: χ^2^ = 8.44, df (1), p = 0.004**  Ndufs4*GF-IL6: χ^2^ = 0.195, df (1), p = 0.659 |
| OB | IL-6 | Mid | **Ndufs4: χ^2^ = 3.96, df (1), p = 0.047**  **GF-IL6: χ^2^ = 5.26, df (1), p = 0.022**  Ndufs4*GF-IL6: χ^2^ = 0.32, df (1), p = 0.857 |
|  |  | Late | Ndufs4: χ2 = 2.12, df (1), p = 0.145  GF-IL6: χ2 = 0.223, df (1), p = 0.637  Ndufs4*GF-IL6: χ2 = 0.443, df (1), p = 0.506 |
|  | TNF-α | Mid | Ndufs4: χ^2^ = 0.537, df (1), p = 0.464  GF-IL6: χ^2^ = 0.008, df (1), p = 0.928  Ndufs4*GF-IL6: χ^2^ = 0.215, df (1), p = 0.643 |
|  |  | Late | **Ndufs4: χ^2^ = 24.79, df (1), p ≤ 0.001**  GF-IL6: χ^2^ = 1.25, df (1), p = 0.264  Ndufs4*GF-IL6: χ^2^ = 0.698, df (1), p = 0.403 |
|  | IL-10 | Mid | Ndufs4: χ^2^ = 0.144, df (1), p = 0.704  GF-IL6: χ^2^ = 0.095, df (1), p = 0.758  Ndufs4*GF-IL6: χ^2^ = 0.289, df (1), p = 0.591 |
|  |  | Late | **Ndufs4: χ^2^ = 17.79, df (1), p ≤ 0.001**  GF-IL6: χ^2^ = 0.002, df (1), p = 0.961  Ndufs4*GF-IL6: χ^2^ = 0.095, df (1), p = 0.758 |

**Figures 4, 5, and 6**

| IBA-1 | Cortex | Mid | Intensity:  Ndufs4: χ^2^ = 3.61, df (1), p = 0.057  **GF-IL6: χ^2^ = 175.03, df (1), p ≤ 0.001**  **Ndufs4*GF-IL6: χ^2^ = 5.0, df (1), p = 0.025** |
| --- | --- | --- | --- |
|  |  |  | IBA-1^+^ cells:  Ndufs4: χ^2^ = 1.83, df (1), p = 0.176  **GF-IL6: χ^2^ = 295.56, df (1), p ≤ 0.001**  **Ndufs4*GF-IL6: χ^2^ = 3.9, df (1), p = 0.048** |
|  |  | Late | Intensity:  **Ndufs4: χ^2^ = 36.85, df (1), p ≤ 0.001**  **GF-IL6: χ^2^ = 241.73, df (1), p ≤ 0.001**  **Ndufs4*GF-IL6: χ^2^ = 46.02, df (1), p ≤ 0.001** |
|  |  |  | IBA-1^+^ cells:  **Ndufs4: χ^2^ = 8.46, df (1), p = 0.004**  **GF-IL6: χ^2^ = 261.38, df (1), p ≤ 0.001**  **Ndufs4*GF-IL6: χ^2^ = 18.61, df (1), p ≤ 0.001** |
|  | CA1 | Mid | Intensity:  **Ndufs4: χ^2^ = 6.06, df (1), p = 0.014**  **GF-IL6: χ^2^ = 252.58, df (1), p ≤ 0.001**  **Ndufs4*GF-IL6: χ^2^ = 6.23, df (1), p = 0.013** |
|  |  |  | IBA-1^+^ cells:  Ndufs4: χ^2^ = 1.99, df (1), p = 0.158  **GF-IL6: χ^2^ = 450.4, df (1), p ≤ 0.001**  **Ndufs4*GF-IL6: χ^2^ = 8.05, df (1), p = 0.005** |
|  |  | Late | Intensity:  Ndufs4: χ^2^ = 0.316, df (1), p = 0.574  **GF-IL6: χ^2^ = 98.33, df (1), p ≤ 0.001**  **Ndufs4*GF-IL6: χ^2^ = 4.48, df (1), p = 0.034** |
|  |  |  | IBA-1^+^ cells:  Ndufs4: χ^2^ = 0.754, df (1), p = 0.385  **GF-IL6: χ^2^ = 219.88, df (1), p ≤ 0.001**  **Ndufs4*GF-IL6: χ^2^ = 19.79, df (1), p ≤ 0.001** |
|  | Cerebellum | Mid | **Ndufs4: χ^2^ = 5.73, df (1), p = 0.017**  **GF-IL6: χ^2^ = 276.2, df (1), p ≤ 0.001**  **Ndufs4*GF-IL6: χ^2^ = 6.25, df (1), p = 0.012** |
|  |  | Late | **Ndufs4: χ^2^ = 10.52, df (1), p = 0.001**  **GF-IL6: χ^2^ = 81-02, df (1), p ≤ 0.001**  **Ndufs4*GF-IL6: χ^2^ = 19.61, df (1), p ≤ 0.001** |
|  | OB | Mid | **Ndufs4: χ^2^ = 20.85, df (1), p ≤ 0.001**  **GF-IL6: χ^2^ = 5.79, df (1), p = 0.016**  Ndufs4*GF-IL6: χ^2^ = 0.568, df (1), p = 0.568 |
|  |  | Late | **Ndufs4: χ^2^ = 35, df (1), p ≤ 0.001**  GF-IL6: χ^2^ = 0.119, df (1), p = 0.73  Ndufs4*GF-IL6: χ^2^ = 3.07, df (1), p = 0.08 |
|  | VN | Mid | Ndufs4: χ^2^ = 3.04, df (1), p = 0.081  **GF-IL6: χ^2^ = 31.6, df (1), p ≤ 0.001**  **Ndufs4*GF-IL6: χ^2^ = 5.38, df (1), p = 0.02** |
|  |  | Late | **Ndufs4: χ^2^ = 29.66 df (1), p ≤ 0.001**  GF-IL6: χ^2^ = 0.715, df (1), p = 0.398  **Ndufs4*GF-IL6: χ^2^ = 7.86, df (1), p = 0.005** |
| GFAP | Cortex | Mid | Ndufs4: χ2 = 3.26, df (1), p = 0.071  **GF-IL6: χ2 = 43.45, df (1), p ≤ 0.001**  Ndufs4*GF-IL6: χ2 = 1.24, df (1), p = 0.266 |
|  |  | Late | **Ndufs4: χ2 = 101.64, df (1), p ≤ 0.001**  **GF-IL6: χ2 = 99.67, df (1), p ≤ 0.001**  **Ndufs4*GF-IL6: χ2 = 57.1, df (1), p ≤ 0.001** |
|  | CA1 | Mid | **Ndufs4: χ2 = 21.89, df (1), p ≤ 0.001**  **GF-IL6: χ2 = 4.74, df (1), p = 0.03**  Ndufs4*GF-IL6: χ2 = 2.97, df (1), p = 0.085 |
|  |  | Late | **Ndufs4: χ2 = 72.58, df (1), p ≤ 0.001**  GF-IL6: χ2 = 1.55, df (1), p = 0.213  **Ndufs4*GF-IL6: χ2 = 27, df (1), p ≤ 0.001** |
|  | Cerebellum | Mid | Ndufs4: χ2 = 0.352, df (1), p = 0.553  **GF-IL6: χ2 = 485.17, df (1), p ≤ 0.001**  Ndufs4*GF-IL6: χ2 = 0.71, df (1), p = 0.4 |
|  |  | Late | Ndufs4: χ2 = 0.23, df (1), p = 0.632  **GF-IL6: χ2 = 154.5, df (1), p ≤ 0.001**  **Ndufs4*GF-IL6: χ2 = 6.1, df (1), p = 0.013** |
|  | OB | Mid | **Ndufs4: χ2 = 55.47, df (1), p ≤ 0.001**  **GF-IL6: χ2 = 4.22, df (1), p = 0.04**  Ndufs4*GF-IL6: χ2 = 0.018, df (1), p = 0.894 |
|  |  | Late | **Ndufs4: χ2 = 157.75, df (1), p ≤ 0.001**  GF-IL6: χ2 = 1.18, df (1), p = 0.276  **Ndufs4*GF-IL6: χ2 = 5.31, df (1), p = 0.021** |
|  | VN | Mid | Ndufs4: χ2 = 0.142, df (1), p = 0.706  **GF-IL6: χ2 = 103.87, df (1), p ≤ 0.001**  Ndufs4*GF-IL6: χ2 = 0.48, df (1), p = 0.489 |
|  |  | Late | **Ndufs4: χ2 = 61.04, df (1), p ≤ 0.001**  **GF-IL6: χ2 = 6.23, df (1), p = 0.013**  Ndufs4*GF-IL6: χ2 = 0.443, df (1), p = 0.384 |
